# Influence of Leptin on the Secretion of Growth Hormone in Ewes under Different Photoperiodic Conditions

**DOI:** 10.3390/ijms24098036

**Published:** 2023-04-28

**Authors:** Maciej Wójcik, Agata Krawczyńska, Dorota Anna Zieba, Hanna Antushevich, Andrzej Przemysław Herman

**Affiliations:** 1 The Kielanowski Institute of Animal Physiology and Nutrition, Polish Academy of Sciences, Instytucka 3, 05-110 Jabłonna, Poland; 2 Department of Nutrition and Animal Biotechnology, and Fisheries, Faculty of Animal Sciences, University of Agriculture in Krakow, 31-120 Krakow, Poland

**Keywords:** leptin, growth hormone, photoperiod, leptin receptor, leptin resistance, growth hormone resistance, anterior pituitary, hypothalamus

## Abstract

Leptin is an adipokine with a pleiotropic impact on many physiological processes, including hypothalamic-pituitary-somatotropic (HPS) axis activity, which plays a key role in regulating mammalian metabolism. Leptin insensitivity/resistance is a pathological condition in humans, but in seasonal animals, it is a physiological adaptation. Therefore, these animals represent a promising model for studying this phenomenon. This study aimed to determine the influence of leptin on the activity of the HPS axis. Two in vivo experiments performed during short- and long-day photoperiods were conducted on 12 ewes per experiment, and the ewes were divided randomly into 2 groups. The arcuate nucleus, paraventricular nucleus, anterior pituitary (AP) tissues, and blood were collected. The concentration of growth hormone (GH) was measured in the blood, and the relative expression of *GHRH*, *SST*, *GHRHR*, *SSTR1*, *SSTR2*, *SSTR3*, *SSTR5*, *LEPR*, and *GH* was measured in the collected brain structures. The study showed that the photoperiod, and therefore leptin sensitivity, plays an important role in regulating HPS axis activity in the seasonal ewe. However, leptin influences the release of GH in a season-dependent manner, and its effect seems to be targeted at the posttranscriptional stages of GH secretion.

## 1. Introduction

Obesity in humans modulates the activity of many physiological pathways at different levels, from the secretion of basic hormones to changes in sensitivity to the hormone’s actions. One such hormone is leptin, a pleiotropic cytokine-like hormone also considered to be involved in the modulation of the hypothalamic-pituitary-somatotropic (HPS) axis activity; however, experimental data from studies examining the role of leptin in HPS axis modulation are ambiguous. This axis is one of the most important hormonal axes and is key in regulating metabolic processes in mammalian organisms. HPS axis activity disturbances, including both lowered and increased activity, affect metabolism, immunology, and reproduction [1]. In rats, leptin has been found to increase growth hormone (GH) secretion both at baseline and after growth hormone-releasing hormone (GHRH) stimulation [2]. Additionally, a study of cow adenohypophyseal explants showed that GH levels were lower after leptin stimulation. It was also demonstrated that the effects of leptin on the HPS axis depend on the nutritional status of the animal [3]. The results emphasize the importance of nutritional status and leptin as a hormone related to HPS axis activity status.

Leptin is mainly secreted by white adipose tissue. Its plasma concentration positively correlates with the amount of adipose tissue in the organism [4]. Leptin regulates feeding behavior and is often called the satiety hormone because it suppresses appetite and increases the metabolic rate primarily by acting through central pathways [4]. This hormone also regulates reproductive function and plays a role in growth processes, proinflammatory immune responses, angiogenesis, and lipolysis [5,6]. It was found that a decrease in tissue sensitivity to leptin leads to the development of obesity and metabolic disorders, such as insulin resistance and dyslipidemia. Mechanisms underlying the development of leptin resistance may include mutations in the gene encoding leptin and its receptors, as well as in proteins involved in the self-regulation of leptin synthesis and blood-brain barrier (BBB) permeability [7].

In seasonal animals, changes in day length are accompanied by changes in many physiological systems [8], including the HPS axis [9] and immunity [10]. A significantly higher GH plasma concentration was observed during the long-day (LD) photoperiod [11,12]. The seasonality of sheep has a crucial impact on how leptin influences these organisms because, during the LD season, natural leptin resistance is observed in these animals [10,13,14,15]. Leptin resistance involves the insensitivity of specific tissues and glands to this hormone and is characterized by hyperphagia, reduced energy expenditure, and hyperleptinemia. The mechanisms of both pathological (in obese humans) and physiological day length-dependent leptin resistance (seasonal animals, e.g., in sheep) are still not fully understood. This natural leptin resistance also seems to influence the HPS axis activity. However, earlier studies indicate that melatonin is an important factor that may act directly on the hypothalamus and change leptin sensitivity. The results showed that both melatonin receptor deficiency and a decrease in the levels of this hormone lead to leptin resistance in mice and rats, respectively [16,17].

Due to the seasonally variable sensitivity of sheep to the action of leptin, it could be hypothesized that the modulatory effect of leptin on HPS axis activity could be photoperiod-dependent. Therefore, this study was performed using a sheep model and was designed to determine the modulatory role of leptin on the secretion of GH and the expression of genes associated with the HPS axis activity in the hypothalamic nuclei, including the arcuate nucleus (ARC) and paraventricular nucleus (PVN), and the anterior pituitary (AP) during short-day (SD) and LD photoperiods.

## 2. Results

### 2.1. The Effect of Leptin and Photoperiod on the Plasma Concentration of GH

**Figure 1:**
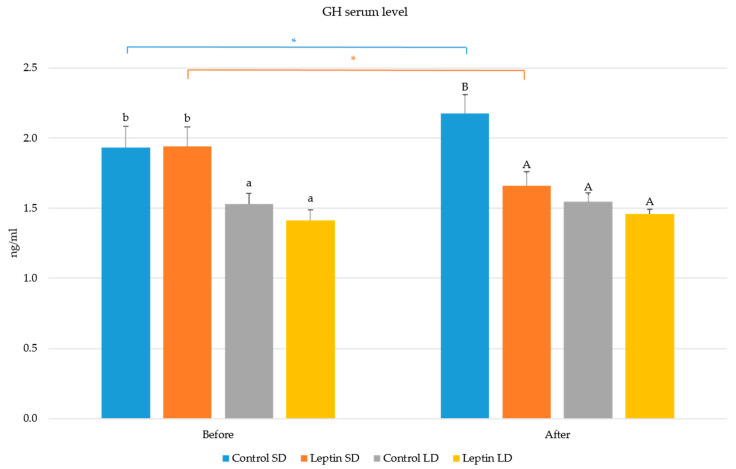
The effect of season and leptin treatment on the plasma GH concentration before and after leptin treatment. GH—growth hormone, SD—short day; LD—long day; * shows the statistically significant differences between before and after treatment with the research factor; Capital letters show differences between the results from each period (separately before and after); lowercase letters refer to statistically significant differences in the period ‘before’; uppercase letters refers to statistically significant differences in the period ‘after’. The ‘before’ results were calculated from the three (means) and the ‘after’—last four (means) time points of blood collection intervals, based on the results obtained from each animal (present in the point chart).

The circulating concentration of GH was lower (*p* < 0.05) during the LD photoperiod than during the SD photoperiod. Injection of leptin reduced (*p* < 0.05) the blood level of GH only during the SD photoperiod, whereas during the LD period, the concentration of GH was not affected by leptin treatment. Moreover, it was found that the circulating concentration of GH increased (*p* < 0.05) in the control ewes during the experiment performed during the SD photoperiod. It is worth noting that time-dependent changes in GH secretion were not observed in the ewes during the LD photoperiod (Figure 1).

### 2.2. Gene Expression

#### 2.2.1. The Effect of Leptin on the Relative Expression of HPS Axis-Associated Genes in the ARC and PVN

*GHRH* mRNA expression in the ARC was higher (*p* < 0.05) during the LD photoperiod. Leptin did not influence the expression of *GHRH* regardless of the photoperiodic condition. Expression of the long form of the leptin receptor (*LEPR)* encoding gene in the ARC was lower (*p* < 0.05) during the LD photoperiod than during the SD period. During both photoperiods, leptin treatment did not change *LEPR* expression. Somatostatin (*SST)* mRNA expression in the PVN was higher (*p* < 0.05) during long days. *LEPR* expression in the PVN did not differ between the photoperiods among the control groups. However, during the SD photoperiod, treatment with leptin caused an increase in *LEPR* expression (*p* < 0.05) in the PVN, while *LEPR* mRNA expression was lower during long days (*p* < 0.05) in the leptin-treated animals than in the control group (Table 1).

#### 2.2.2. The Effect of Leptin on the Relative Expression of HPS Axis-Associated Genes in the AP

Growth hormone-releasing hormone receptor (*GHRHR)* mRNA expression did not differ between photoperiods among the control groups. The expression of *GHRHR* was also not influenced by leptin treatment, regardless of the season. Somatostatin receptor (*SSTR) 1* mRNA expression was higher (*p* < 0.05) during short days in the control groups. Leptin treatment did not influence the expression of *SSTR1* regardless of the season. *SSTR2* expression was higher during the LD photoperiod (*p* < 0.05). Leptin treatment increased *SSTR2* expression only during the LD photoperiod (*p* < 0.05). *SSTR3* expression did not differ between the photoperiods among the control groups. Leptin treatment did not cause any difference in *SSTR3* expression during either photoperiod. Differences in *SSTR5* expression were not observed between the photoperiods among the control groups. Additionally, leptin treatment did not influence *SSTR5* expression regardless of the photoperiod. *GH* mRNA expression was higher during the SD photoperiod (*p* < 0.05) than during the LD period in the control group. Leptin treatment did not influence the expression of *GH* regardless of the photoperiod (Table 2). *LEPR* mRNA expression was higher during the SD photoperiod; however, administration of leptin did not affect the mRNA expression of this gene.

## 3. Discussion

The results of the present study showed that intravenous (i.v.) administration of leptin had an inhibitory effect on GH release during the SD photoperiod; however, leptin treatment did not influence the circulating concentration of GH during the LD period in sheep [3]. It is worth mentioning that an in vivo study on rodents suggested that leptin has a stimulatory effect on GH secretion. An experiment investigating male rats showed that intracerebroventricular (ICV) infusion of leptin stimulated both spontaneous pulsatile GH secretion and the GH response to GHRH [2], and a study investigating leptin-deficient obese mice (ob/ob) showed that these animals were characterized by decreased circulating GH concentration and pituitary GH and ghrelin receptor (GHS-R) mRNA levels, whereas hypothalamic *GHRH* and *SST* expression in these animals did not differ from that in lean controls [18]. A study on castrated estrogen-treated yearling male sheep found that leptin administration increased GH pulse amplitudes and mean concentrations of circulating GH [19]. Since we did not measure pulsatile GH release, the results of the current study cannot be compared to the mentioned results regarding pulsatile GH secretion. However, basal GH secretion is the most studied in other studies, and almost all of our experiments, and this information is sufficient to show the effect of leptin on GH secretion. These results suggest that the effects of leptin on GH secretion could also be dependent upon the species, sex, and/or hormonal status of an animal. Generally, the effect of leptin on GH secretion is still elusive and seems to also be dependent upon the use of an in vivo vs. an in vitro experimental model. An in vitro study on primary cultured ovine somatotrophs showed that, although short-term leptin treatment did not influence GH release, long-term leptin treatment suppressed GH secretion [20]. Moreover, the positive effect of leptin on GHRH-induced GH release has been reported in studies of primary monolayer cultures of pituitary cells in rats [21]. However, opposite results have been published by Roh et al. (2001) [22], who observed that leptin has a negative effect on GH secretion stimulated by GHRH from primary sheep cell culture. The results of an in vitro study on cultured GH3 cells showed that leptin inhibited the basal GH secretion of GH3 cells, and it was suggested that the inhibitory effect of leptin on GH synthesis and secretion might be related to the decreasing level of intracellular free Ca(2+) [23]. An ex vivo study on bovine AP explants showed that leptin could directly act on the AP to modulate GH release, and this effect was dependent upon the fasted vs. normal-fed nutritional history of the cattle [3]. It was found that higher doses of leptin led to lower basal GH secretion than control treatment in tissues collected from normal-fed cows. However, GH release from AP explants from fasted cows treated with the lowest dose of leptin was higher than that from control explants, but larger doses had no effect. Moreover, leptin caused an inversely related, dose-dependent increase in GHRH-mediated GH release in tissues from normal-fed cows. These results may have been due to differences in physiology between somatotrophs maintained for a short time (explants) and the long-term (primary) culture [24].

Our results obtained in ewes from the present experiment suggest that the effect of leptin on GH secretion is highly dependent upon the photoperiodic condition. It is worth noting that the leptin–basal concentration was at a significantly higher level during the LD period [25]. The higher concentration of leptin in plasma was also maintained at every time point of the experiment in the group treated with leptin [25]. These results are consistent with those reported in the paper by Marie et al. (2001) [26]. She showed that during the LD season, the concentration of leptin in blood plasma was 180% increased than during the SD season, but this change was not associated with the anorectic action of leptin. During this period, when readily accessible food is abundant, sheep exhibit increased appetite and appear to be insensitive to the high concentrations of leptin that result from increased adiposity. Seasonal leptin resistance allows these animals to live in changing climates and store energy that they can use during periods of reduced food availability. During the autumn and winter, sheep exhibit physiological sensitivity to leptin, and their appetite adjusts in proportion to their nutritional status [13]. In the present experiments, leptin treatment failed to influence GH secretion during the LD season when administered using the same dose as that used during the SD period. This finding suggests that some type of leptin resistance in the pituitary occurs during the SD photoperiod. Similar observations were made by Szczesna et al. (2011) [27]. This group reported on the coexistence of leptin resistance in the pituitary with the maintenance of leptin sensitivity in the hypothalamus based on SOCS3 expression during SD seasons. Although many mechanisms may cause leptin resistance, one of them is certainly the lower sensitivity of cells/tissues to the action of leptin associated with the reduction in the expression of its receptor, which is responsible for leptin signal transduction; this resistance is also accompanied by an increased concentration of leptin in the blood. The results of those experiments demonstrated that leptin infuses centrally into the third brain ventricle, affecting the expression of SOCS3 factors in a seasonal and tissue-dependent manner. These results suggested that central leptin infusion alters the expression of SOCS3 factors in sheep, which leads to alterations in the hypothalamic and pituitary sensitivity to the actions of leptin [13]. The importance of LEPR in regulating AP function was suggested by studies that showed the prevalence of both long and common forms of mRNA and leptin receptor protein in normal AP cells [28]. Our experiment showed that long-day ewes are characterized by significantly lower gene expression of the LEPR gene in the AP than short-day ewes. Lower expression of the LEPR gene in the AP may explain the lack of effect of leptin treatment on the circulating concentration of GH in long-day ewes. It is worth mentioning that long-day ewes showing lower LEPR gene expression in the AP were also characterized by lower circulating concentrations of GH. This result supports the result of a study on rodents that reported that mice with the deletion of a long LEPR isoform showed a substantial decrease in the circulating concentration of GH [29]. It is worth mentioning that the occurrence of physiological LEP resistance has been reported in seasonal animals, including sheep [13].

Importantly, leptin could influence the secretion of GH not only by directly influencing somatotropic cells in the pituitary gland but also by interfering with the hypothalamic nuclei involved in the control of GH secretion. Although thus far it is not entirely clear whether leptin has to penetrate brain barriers to exert its central effects, it is generally accepted that brain access is important for leptin to exert its physiological actions. Due to its size (16 kDa), leptin seems unable to diffuse passively through brain barriers. However, it has been previously reported that leptin has direct access to circumventricular organs, including LEPR-expressing neurons in the mediobasal hypothalamus that are not shielded by the BBB [30,31]. The existence of differential methods of regulation of leptin transport in the central nervous system (CNS) via the choroid plexus and the BBB has also been suggested. Specific high-affinity transport systems for leptin in the hypothalamus and across the choroid epithelium of the blood-cerebrospinal fluid (CSF) barrier seem to play a key role in regulating leptin entry into the CNS and CSF under physiological conditions. At higher pharmacological concentrations of leptin and over a longer period, transport via the BBB takes over [32]. Moreover, another study suggests that intact leptin could be partially transported from blood to the brain by a saturable system [33]. Leptin could also be transported by tanycytes within the mediobasal hypothalamus; however, this requires the expression of LEPR [34]. Moreover, a study on mice with inducible deletion of LEPR in brain endothelial and epithelial cells (LepRbeKO) showed that leptin uptake by the brain depends on the presence of LEPR in brain barriers [31]. The important role of LEPR isoforms in leptin uptake in the brain was also suggested in a study conducted on mice lacking all LEPR isoforms. In these animals, leptin uptake by the brain was significantly reduced [35]. However, the results of another study on a rat strain deficient in all LEPR isoforms showed that the expression of the LEPR gene was not important for leptin uptake in the brain [36]. Moreover, GH could enable leptin to act on metabolism. The influence of GH on metabolism was reported to occur in a manner that depends on leptin, among other hormones, and conditions that affect glucose homeostasis, including insulin sensitivity, appetite, energy expenditure, and neuroendocrine adaptation in response to different forms of metabolic stress, such as glucose deprivation, food restriction, and physical exercise [37,38]. Additionally, our results confirm that food deprivation increases the circulating concentration of GH in sheep. However, this effect was observed only during the SD photoperiod. The stimulating effect of food deprivation was stated before in both ruminants and humans [39]. Moreover, the study on sheep under long-term metabolic manipulation, which led to creating lean and fat animals, showed that the leptin concentration was significantly higher and GH concentration was higher in fat animals [40]. These season-dependent influences of food deprivation on the GH concentration may further support our results, suggesting that leptin modulates GH secretion in sheep only during the SD photoperiod. Since the sheep in this experiment were not fed immediately before and during the experiment (more than 12 h of food deprivation), it is possible there was a decrease in endogenous leptin production and, thus, a decrease in the inhibitory effect of this hormone on GH secretion in the pituitary gland.

Our study showed that LEPR is expressed both in the ARC and PVN, the regions of the hypothalamus involved in the central control of GH secretion from the pituitary. The pattern of LEPR gene expression in the ARC and AP was similar. This finding may suggest that the reduced sensitivity of the AP hypothalamic nucleus to leptin action occurs during the LD photoperiod. However, the results of our research showed different patterns for both seasonal and leptin treatment responses. In the PVN, *LEPR* mRNA expression did not appear to be affected by the length of the day, while the seasonally dependent effect of leptin treatment on the expression of this gene was demonstrated. This regulatory effect of leptin on *LEPR* mRNA expression in the PVN may be related to its role in regulating the activity of the sympathetic nervous system, in which leptin has been shown to play an important role [41,42]. Therefore, the decrease in *LEPR* mRNA expression during long days could act as a mechanism that enables adaptation to the physiological increase in leptin concentration during this period.

Moreover, our study showed that peripheral administration of leptin did not influence either *GHRH* or *SST* expression in the hypothalamus. These results are generally consistent with a previous experiment on mice, in which peripherally administered leptin did not alter hypothalamic *GHRH* and *SST* mRNA levels compared with vehicle-treated controls [18]. Additionally, in cows treated intracerebroventricularly with leptin, no changes in GHRH secretion into the CFS were found [43]. However, a study on rats showed that central leptin injection increased *GHRH* mRNA levels and reduced *SST* mRNA levels in the hypothalamus [44]. Interestingly, *GHRH* expression was higher during the LD photoperiod than during the SD period, and this observation was not consistent with the changes in the circulating concentration of GH. However, the previously mentioned study on cows also reported a weak correlation between the concentrations of GHRH in the CSF and serum concentrations of GH [43]. This could suggest that the photoperiodic condition influences GH secretion via mechanisms independent of the hypothalamic GH-regulatory hormones. However, the action of hypothalamic GH-regulatory hormones in the AP depends upon the expression of their corresponding receptor in pituitary cells. Our study suggests that the expression of GHRHR in the AP is generally constant and not influenced by leptin treatment or photoperiodical conditions, but the same cannot be said for the expression of the SST receptor. Earlier studies showed that SSTR2 and SSTR5 are two receptors that play an essential role in SST inhibitory action in HPS axis activity [45]. Research on rats showed that *Sstr5* gene expression was confirmed in approximately 86% of somatotrophs, while *Sstr2* expression was measured in approximately 42% of these cells [46]. Our research showed that during the SD season, when the GH serum concentration was higher, the expression of *SSTR2* was more than 5-fold lower than during the LD photoperiod. It was previously found that SST signals predominantly through SSTR2, and the activation of this receptor via intact adenylate cyclase and mitogen-activated protein kinase systems mediates the inhibitory actions of SST [45,47]. Moreover, we found that the expression of *SSTR5* mRNA in the AP was increased during the LD photoperiod as well as in SD conditions in animals treated with leptin. This finding may partially explain the reduced secretion of GH in ewes injected with leptin during the SD photoperiod. The *SSTR5* gene is expressed in GH-releasing cells and plays a critical role in the physiological regulation of GH secretion [48]. However, it should be mentioned that research conducted on mice showed that the lack of *Sstr2* and *Sstr5* expression did not lead to an elevation in serum GH concentration, which may suggest a compensatory function of SSTR1 and SSTR3 [49,50,51,52]. Our study showed that leptin and photoperiodic conditions did not influence the gene expression of *SSTR3*; however, it was determined that, unlike *SSTR2* and *SSTR5,* the expression of *SSTR1* mRNA in the AP was suppressed during the LD photoperiod. Similar to the rest of the SST receptors, SSTR1 is involved in modulating GH secretion [53]. However, SSTR1 is expressed mainly in the gastrointestinal tract [54,55] and in brain tissue, and its activity could be masked by the action of SSTR2 and SSTR5, which are predominantly expressed in brain and endocrine cells. Therefore, the changes in *SSTR1* expression may be less important for the sensitivity of the AP to SST action.

## 4. Material and Methods

### 4.1. Animals

Experiments were conducted on 24 2–3-year-old Polish Blackface ewes during different photoperiods: LD (May/June) and SD (November/December). During each photoperiod, the animals were randomly divided into two groups (6 ewes in each group):

- Control—intravenously treated with 0.9% *w/v* NaCl (Baxter, Deerfield, IL, USA) in a volume equal to that used in the experimental group;
- LEP—intravenously injected with ovine recombinant leptin (Protein Laboratories Rehovot (PLR) Ltd., Rehovot, Israel) at a dose of 20 µg/kg body weight suspended in saline (0.9% *w/v* NaCl, Baxter, Deerfield, IL, USA). The leptin dose was selected based on the dose used by Maciel et al. [56] for growing beef heifers.

During the experiments, the ewes were allowed to maintain eye contact and exposed to natural daylight and exposed to natural daylight. The ewes were fed commercial concentrates and hay and had ad libitum water access. Before the experiments, the estrous cycles of the ewes during the SD period were synchronized using a method based on intravaginal sponges—Chronogest^®^ CR (Merck Animal Health, Amsterdam, The Netherlands) according to a previously described protocol [57]. Twenty-four hours before the experiment began, the animals were treated with 500 IU of pregnant mare serum gonadotropin (PMSG) (Merck Animal Health, Amsterdam, The Netherlands). During the LD period, the synchronization of animals was not performed because the ewes were in seasonal anestrous. At 12 h before the start of the experiments, the animals were deprived of food. Blood collection started 1.5 h before the leptin injection. Blood was collected in 30-min intervals until the end of the experiment. During the calculations, the results from the first three time points and the last four time points of blood collection were obtained from each animal.

The experiments lasted 4 h (1.5 h before followed by 2.5 h after leptin treatment), and then the animals were euthanized using a pharmacological agent, Morbital^®^ (Biowet Puławy Sp. z.o.o; Puławy, Poland), which was administered intravenously at a dose of 80 mg/kg of body mass, and the AP and hypothalamic tissues were collected and snap frozen in liquid nitrogen. Until further analysis, the tissues were stored at −80 °C.

The experimental procedures were approved (authorization no. 56/2013 from 23 October 2013) by the 3rd Local Ethics Committee of the Warsaw University of Life Sciences—SGGW (Warsaw, Poland).

### 4.2. Real-Time (RT)-PCR Assay

Total RNA from collected tissues was isolated using a NucleoSpin RNA/Protein kit (Macherey-Nagel, Dueren, Germany). The quality and quantity of the RNA were verified using a NanoDrop ND-1000 spectrophotometer (Thermo Fisher Scientific, Waltham, MA, USA) at wavelengths of 260, 280, and 230 nm and using 1% agarose gel electrophoresis. Subsequently, RNA was transcribed using a reverse transcription Maxima™ First Strand cDNA Synthesis Kit for Real-Time Quantitative Polymerase Chain Reaction (RT-qPCR) (Thermo Fisher Scientific, Waltham, MA, USA). The obtained matrix was used for real-time PCR using the FIREPol^®^ HOT EvaGreen qPCR Mix^®^ Plus kit (Solis Biodyne, Tartu, Estonia). The temperature profile for the amplification of each gene was chosen based on an optimized standard protocol: 95 °C for 15 min for HOT FIREPol^®^ DNA polymerase activation and 40 cycles at 95 °C for 5–10 s for denaturation, 60 °C for 10–30 s for annealing, and 72 °C for 15–30 s for the extension. The primers used for each measured gene are presented in Table 3. The amplified products of the originally designed primers were sequenced to confirm their correct design. The PCR experiments were carried out using a thermocycler RotorGene Q (Qiagen, Germantown, MD, USA) with RotorGene Q software. The results obtained for the measured genes were normalized to the levels of the reference gene or the combination of the reference genes, including glyceraldehyde 3-phosphate dehydrogenase (GAPDH), β-actin (ACTB), histone deacetylase 1 (HDAC1), beta-2-microglobulin (*B2M*), and cyclophilin C (*PPIC*), using the NormFinder (Molecular Diagnostic Laboratory, Aarhus University Hospital, Aarhus, Denmark) program to identify of the optimal normalization gene. The results are presented in arbitrary units as the ratio of the expression of the target gene to the expression of the reference gene and the mean gene expression in the SD was set as 1 [58].

### 4.3. Radioimmunological Assay

The GH concentration in plasma was measured by the radioimmunological assay (RIA) double-antibody method using anti-bovine GH and anti-rabbit γ-globulin antisera and a bovine GH standard (NIDDK-GH-B-1003A). The characteristics of the antiserum and method were comprehensively described by Dvorak et al. [62]. The assay’s sensitivity for GH was 0.6 ng/mL, and the intra- and interassay coefficients of variation were 5.9 and 10.2%, respectively.

### 4.4. Statistical Analysis

The statistical analysis was performed using TIBCO Statistica 13.3 (TIBCO Statistica Ltd., Palo Alto, CA, USA). The significant differences in gene expression between the experimental groups were determined using a two-way analysis of variance (ANOVA) with the following factors: season or leptin treatment. The significance of the differences in blood GH concentration was determined using two-way ANOVA (comparison among the groups before or after treatment) or a Student’s *t*-test for dependent samples (comparison between before vs. after for each group separately). Each ANOVA was followed by Fisher’s post hoc test, and results *p* ≤ 0.05 were deemed statistically significant. Before each ANOVA, the normality (Shapiro-Wilk’s test) and variance homogeneity (Levene’s test) were evaluated. If the ANOVA assumptions were violated, the data were log-transformed. The data are presented as the mean ± standard error of the mean (SEM).

## 5. Conclusions

Our study shows that leptin influences the release of GH in a season-dependent manner, and its effect seems to be targeted at the posttranscriptional stages of GH secretion. The lack of effect of leptin treatment on GH secretion during the LD photoperiod may result from the suppressed expression of the gene encoding LEPR in the AP, which suggests that, during the LD photoperiod, leptin resistance occurs in this gland. Reduced expression of *LEPR* was also found in the ARC, while in the PVN, changes in *LEPR* expression in response to leptin treatment may suggest that seasonal changes in sensitivity to leptin occur in the hypothalamus. However, the lack of leptin-induced changes in the expression of *GHRH* and *SST*, regardless of the photoperiodic conditions, indicates that the effect of leptin on GH secretion is dependent upon changes in the secretion of hypothalamic GH regulatory hormones. Our findings suggest that the mechanism by which both leptin and photoperiod influence GH secretion in the AP is dependent upon changes in the sensitivity of pituitary cells to the actions of SST.

## Figures and Tables

**Table 1:** The relative expression of growth hormone-releasing hormone (GHRH), leptin receptor (LEPR), and somatostatin (SST) genes in hypothalamic structures, including the arcuate nucleus and paraventricular nucleus. Letters indicate significant differences between the groups, and the results are presented in arbitrary units.

Hypothalamic Structure	Gene	Control SD	Leptin SD	Control LD	Leptin LD
Arcuate nucleus (ARC)	*GHRH*	1 ± 0.08 ^A^	1.2 ± 0.04 ^A^	1.54 ± 0.11 ^B^	1.72 ± 0.14 ^B^
	*LEPR*	1 ± 0.24 ^B^	0.95 ± 0.17 ^B^	0.58 ± 0.03 ^A^	0.66 ± 0.09 ^A^
Paraventricular nucleus (PVN)	*SST*	1 ± 0.09 ^A^	1.17 ± 0.15 ^AB^	1.81 ± 0.4 ^B^	1.3 ± 0.19 ^AB^
	*LEPR*	1 ± 0.13 ^B^	1.31 ± 0.11 ^C^	0.91 ± 0.03 ^B^	0.6 ± 0.08 ^A^

**Table 2:** The relative gene expression of the following proteins: growth hormone-releasing hormone receptor (GHRHR), somatostatin receptor 1 (SSTR1), somatostatin receptor 2 (SSTR2), somatostatin receptor 3 (SSTR3), somatostatin receptor 5 (SSTR5), growth hormone (GH), and leptin receptor (LEPR) in the ovine anterior pituitary. Letters indicate significant differences between the groups, and the results are presented in arbitrary units.

Gene	Anterior Pituitary			
	Control SD	Leptin SD	Control LD	Leptin LD
*GHRHR*	1 ± 0.06	0.92 ± 0.1	0.90 ± 0.10	1.00 ± 0.08
*SSTR1*	1 ± 0.07 ^B^	1.19 ± 0.13 ^B^	0.37 ± 0.03 ^A^	0.43 ± 0.05 ^A^
*SSTR2*	1 ± 0.32 ^A^	0.69 ± 0.13 ^A^	5.45 ± 0.77 ^B^	7.51 ± 0.61 ^C^
*SSTR3*	1 ± 0.08	0.90 ± 0.07	1.01 ± 0.09	1.03 ± 0.04
*SSTR5*	1 ± 0.08	0.90 ± 0.07	1.01 ± 0.09	1.03 ± 0.04
*GH*	1 ± 0.06 ^B^	1.05 ± 0.03 ^B^	0.81 ± 0.06 ^A^	0.7 ± 0.05 ^A^
*LEPR*	1 ± 0.19 ^B^	1 ± 0.12 ^B^	0.57 ± 0.14 ^A^	0.55 ± 0.08 ^A^

**Table 3:** Primer descriptions.

Gene Symbol	Primer		Gene Bank Accession Number	References
	Forward	Reverse		
*GH*	TTCCTCAGCAGAGTCTTCACC	GGGGTAACATCTTCCAGCTC	XM_027973953.1	Originally designed
*GHR*	ACTGTTAGCCCAAGTATTCC	ATATGGCAAGTTCAGTGAGG	XM_012096677.3	Originally designed
*GHRH*	CCTCTCAGGATTCCACGGTA	CGTACCTTTGCTCCTTGCTC	XM_027976451.1	Originally designed
*GHRHR*	CTTCTCTCACTTCAGCTTGG	GGATTTCTCCTTCAGTCAGC	NM_001009454.3	Originally designed
*SST*	CTCCATCGTCCTGGCTCTT	AGTACTTGGCCAGTTCCTGTTT	XM_027966037.1	Originally designed
*SSTR1*	ACTCCATGGTCATCTACGTG	GAAGCAATGTGGAGGTGAC	XM_012098844.3	Originally designed
*SSTR2*	TCTCTCTGCTGGTCATCTTG	CGTAGATGATGAACCCTGTG	XM_004013144.4	Originally designed
*SSTR3*	CACTGGTCTATCTGGTGGTG	TTGAGGATGTAGACATTGGTG	XM_004006732.4	Originally designed
*SSTR5*	TGGTCATCTATGTGGTCCTG	AGTAGGAGATGGCGTTTTG	NM_001009265.1	Originally designed
*LEPR*	CTGTGCCAACAGCCAAACT	GTGGATCAGGCTTCACAACA	NM_001009763.1	[59]
*ACTB*	GCCAACCGTGAGAAGATGAC	TCCATCACGATGCCAGTG	NM_001009784.2	[60]
*GAPDH*	TGACCCCTTCATTGACCTTC	GATCTCGCTCCTGGAAGATG	NM_001190390.1	[61]
*HDAC1*	CTGGGGACCTACGGGATATT	GACATGACCGGCTTGAAAAT	XM_004005023.3	[58]
*PPIC*	TGGCACTGGTGGTATAAGCA	GGGCTTGGTCAAGGTGATAA	XM_004008676.5	[61]
*B2M*	CTTCTGTCCCACGCTGAGTT	GGTGCTTAGAGGTCTCG	XM_012180604.3	Originally designed

## Data Availability

The data presented in this study are available upon request from the corresponding author.
